# Diagnostic challenges and laparoscopic management of mitotically active cellular fibroma of the ovary: a case report and literature review

**DOI:** 10.3389/fonc.2025.1680968

**Published:** 2025-10-15

**Authors:** Shuxu Tian, Juan Hao, Yulu He, Xuan Liu, Huangbei Song, Chenchen Geng

**Affiliations:** ^1^ Department of Gynecology, Qingdao Women and Children’s Hospital, Qingdao University, Shandong, Qingdao, China; ^2^ Department of Pathology, Peking University People’s Hospital, Qingdao; Qingdao Women and Children’s Hospital, Qingdao University, Shandong, Qingdao, China; ^3^ Department of Ultrasound, Qilu Hospital of Shandong University (Qingdao), Shandong, Qingdao, China

**Keywords:** ovary, mitotically active cellular fibroma, fibrosarcoma, laparoscopy, case report

## Abstract

Mitotically active cellular fibroma (MACF) is a rare ovarian neoplasm characterized by mild to moderate cytological atypia and increased mitotic activity, often presenting diagnostic challenges due to its overlap with ovarian fibrosarcoma. Although MACF has low malignant potential and generally confers a favorable prognosis, accurate diagnosis is essential to avoid overtreatment and to guide appropriate management. We retrospectively analyzed a 33-year-old woman with a 5cm left ovarian mass showing abundant vascularity on ultrasonography. Pathological examination revealed a cellular fibroma with moderate atypia, a high mitotic rate (15/10 High-Power Fields [HPF]), and a Ki-67 index of 60%, creating diagnostic ambiguity with fibrosarcoma. Following multidisciplinary review and adherence to World Health Organization (WHO) criteria, a diagnosis of MACF was confirmed. The patient underwent laparoscopic unilateral salpingo-oophorectomy, with en bloc removal of the tumor. Her postoperative course was uneventful, and no recurrence was observed after over two years. This case underscores that high proliferative indices alone are insufficient for a fibrosarcoma diagnosis and highlights the safety and efficacy of minimally invasive, fertility-sparing surgery for MACF in young patients.

## 1 Introduction

Mitotically active cellular fibroma (MACF) of the ovary is a rare and distinctive subtype of ovarian fibroma, characterized by high cellularity, mild to moderate cytological atypia, and increased mitotic activity (≥4 mitoses per 10 high-power fields [HPF]). MACF is classified as a borderline tumor with low malignant potential, but it carries a small risk of recurrence. Its pathological features often overlap with those of ovarian fibrosarcoma, a highly aggressive malignancy, making the differential diagnosis challenging. An accurate diagnosis is therefore essential to prevent patient overtreatment while ensuring appropriate management. Here, we present a case that exemplifies this diagnostic challenge, particularly in the context of high proliferative markers, and demonstrates the successful application of a conservative, minimally invasive surgical approach.

## 2 Case report

Written informed consent for publication of this case and accompanying images was obtained from the patient.

**Figure 1:**
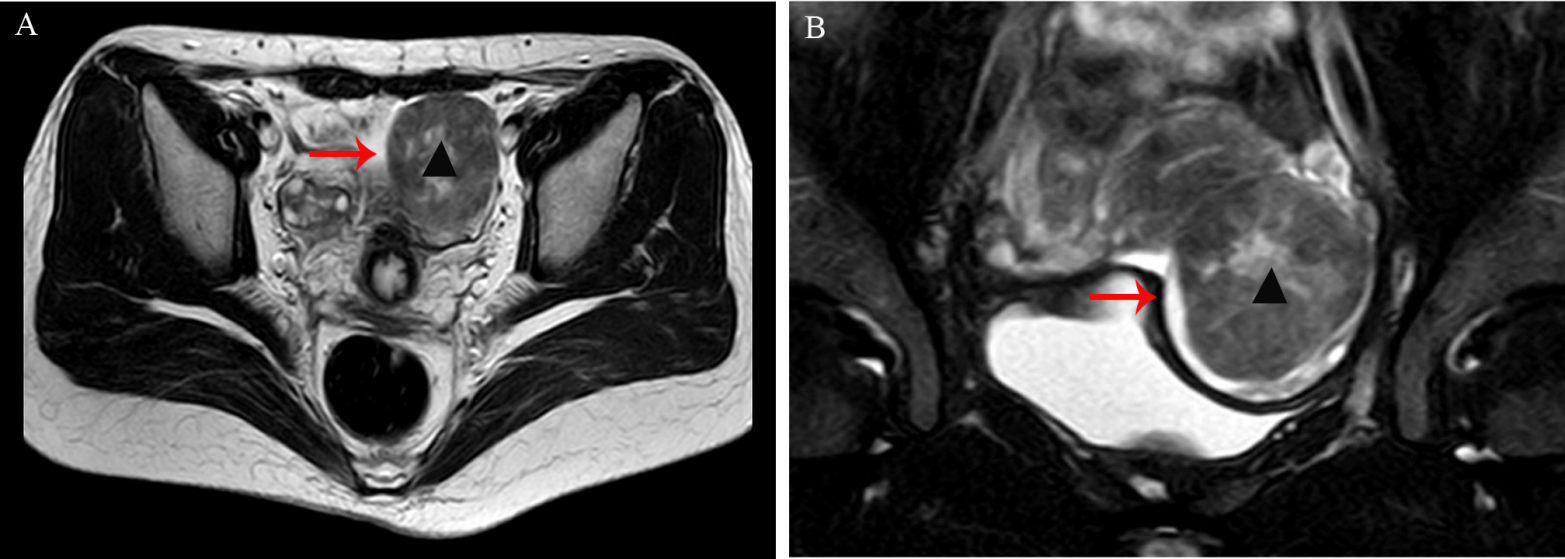
**(A, B)** Pelvic magnetic resonance imaging (MRI) revealed a well-circumscribed mass anterolateral to the uterus, measuring 49×51×55mm, with mildly reduced T2 signal intensity (arrow, indicated by ▴).

A 33-year-old woman (gravida 1, para 1) was found to have a solid mass in the left adnexal region during an incidental examination two years earlier; she remained asymptomatic throughout this period. Initial ultrasonography demonstrated a hypoechoic mass within the left ovary measuring approximately 35×31mm, with well-defined margins, heterogeneous internal echogenicity, and peripheral linear vascular signals. Serial monitoring revealed progressive enlargement of the tumor to 53×48mm, accompanied by increased vascularity (resistance index [RI]: 0.53). Pelvic magnetic resonance imaging (MRI) revealed a well-circumscribed mass anterolateral to the uterus, measuring 49×51×55mm, with mildly reduced T2 signal intensity (**Figure 1**). Serum tumor markers, including CA125, HE-4, CA19-9, AFP, and CEA, were all within normal reference ranges.

**Figure 2:**
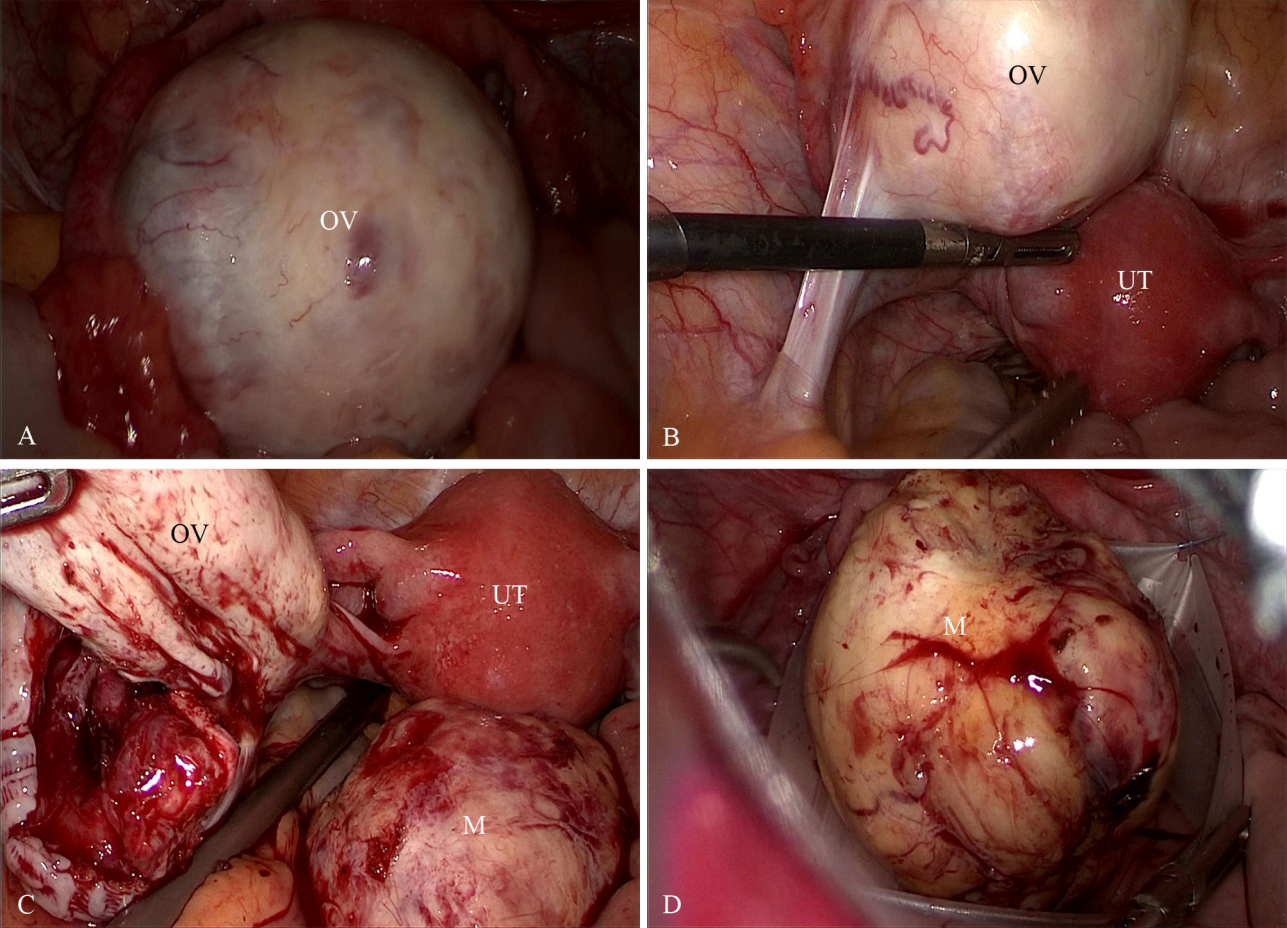
**(A, B)** Laparoscopic images show an enlarged left ovary containing a 5cm solid mass. The mass was pale-yellow, soft in consistency, had an intact ovarian cortex, and exhibited a highly vascularized surface. **(C, D)** The tumor was completely excised and extracted in an endoscopic specimen retrieval bag. OV, ovary; UT, uterus; M, the ovarian mass.

A standard three-port laparoscopy was performed with a 10mm umbilical camera port and two 5-mm accessory ports in the left lower quadrants. Pneumoperitoneum was established to 12mmHg. Laparoscopic exploration revealed an enlarged left ovary containing a 5cm solid mass. The mass was pale-yellow, soft in consistency, had an intact ovarian cortex, and exhibited a highly vascularized surface. The mass was excised with an intact capsule. The specimen was placed into a retrieval bag (KANGJI ™/Type A set, with an opening width of 80mm) and extracted transumbilically by extending the umbilical fascial incision to 20mm (**Figure 2**). No morcellation was used, and there was no intraoperative rupture or spillage. Intraoperative frozen section revealed a “spindle-cell neoplasm with high cellularity and mitotic activity, suspicious for a sex cord-stromal tumor; malignancy cannot be definitively excluded”. Critically, the pathologist did not identify definitive features of malignancy such as severe, diffuse nuclear atypia, atypical mitoses, or tumor necrosis. In the absence of these overt malignant features, and considering the tumor’s encapsulated appearance, the multidisciplinary intraoperative decision was that immediate surgical staging was not warranted. Therefore, the preoperatively planned fertility-sparing unilateral salpingo-oophorectomy was completed. If malignancy had been suspected on frozen section, our plan was to consider conversion to laparotomy for comprehensive staging. Based on the frozen-section impression and in accordance with the patient’s preoperative consent, a left salpingo-oophorectomy was performed to achieve definitive local control while preserving fertility.

**Figure 3:**
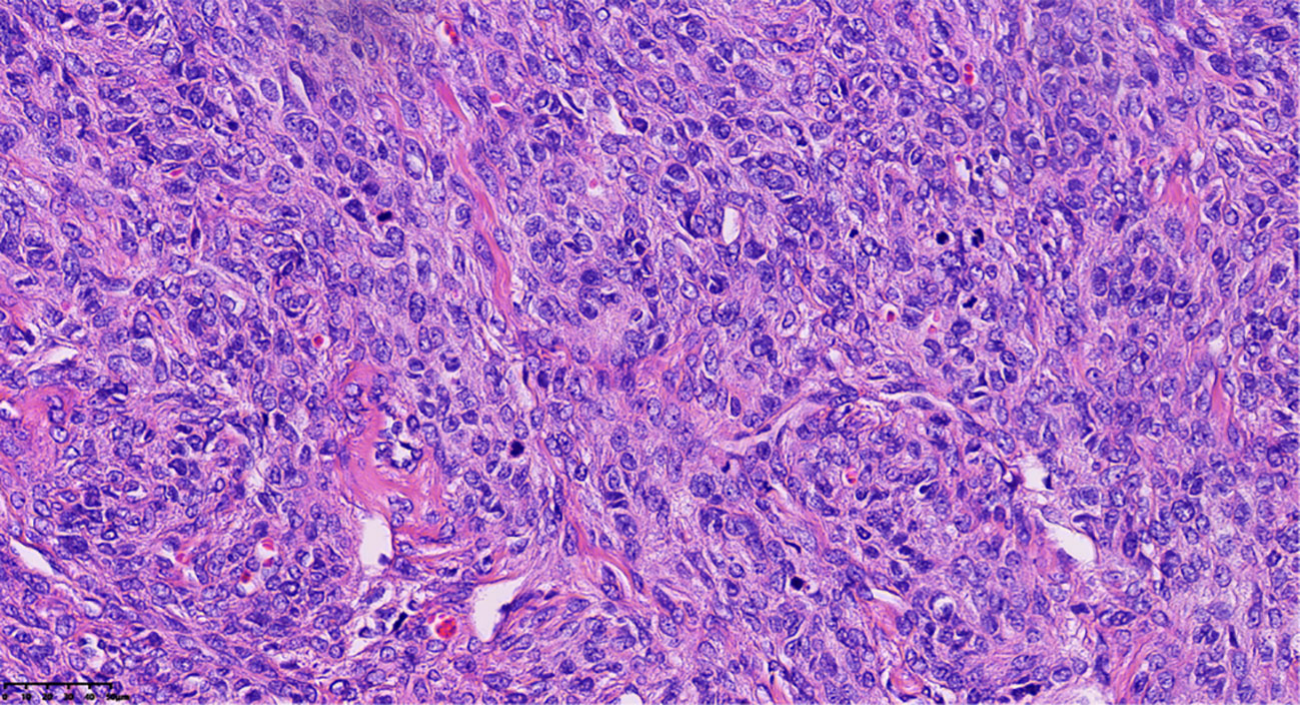
Pathological clues of tumor cells revealed densely cellular spindle cell tumor areas, with foci of moderate cytological atypia and pronounced mitotic activity, approximately 15/10 HPF (Hematoxylin and eosin stain; magnification, ×40).

Final histopathological examination revealed a densely cellular spindle cell tumor. Mitotic figures were counted in the areas of highest cellularity (“hot spots”) across 10 consecutive HPFs using a 40x objective (ZEISS™/Lab.A1, field area 0.237 mm²), revealing a pronounced mitotic rate of approximately 15/10 HPF (equivalent to 6–7 mitoses/mm²). Foci of moderate cytological atypia were present; however, diffuse severe atypia, atypical mitoses, and tumor necrosis were explicitly sought and found to be absent (**Figure 3**). These features, together with the clinical course, supported MACF rather than fibrosarcoma despite the brisk mitotic activity. Immunohistochemical (IHC) staining showed tumor cell positivity for Vimentin (Vim) (confirming mesenchymal origin) and CD99, with focal expression of Smooth Muscle Actin (SMA) and the sex-cord stromal markers α-inhibin and calretinin. Staining was negative for Desmin and Caldesmon (ruling out smooth muscle origin), Cytokeratin 7 (CK7) and Epithelial Membrane Antigen (EMA) (ruling out epithelial origin), and the germ cell tumor markers Wilms Tumor 1 (WT1), Spalt-like transcription factor 4 (SALL4), and Octamer-binding transcription factor 4 (OCT4). This immunoprofile was most consistent with a sex cord-stromal tumor of fibroblastic type. The Ki-67 labeling index was markedly elevated at approximately 60%.

Notably, due to the high mitotic index and proliferative activity, there was initial debate among expert pathologists regarding the diagnosis of fibrosarcoma. The case was reviewed by two senior gynecologic pathologists and underwent external consultation at a national tertiary pathology center (Peking University People’s Hospital, Beijing/China). The final diagnosis of MACF was made based on the 2020 WHO classification criteria, which stipulate that a diagnosis of fibrosarcoma requires the presence of both high mitotic activity and significant (diffuse, moderate-to-severe) nuclear atypia. The absence of these key malignant features, despite the high mitotic rate, was decisive in excluding fibrosarcoma and obviated the need for more radical surgery. The patient’s postoperative course was uneventful. Her surveillance schedule consisted of clinical examinations and transvaginal ultrasonography at 3, 6, 12, 18, and 24 months post-surgery; CT was checked at 12 and 24 months. She remains free of recurrence at over two years of follow-up, with all evaluations negative for disease.

## 3 Discussion

Ovarian fibroma is the most prevalent benign sex cord-stromal tumor (SCST), almost entirely composed of fibroblasts, accounting for approximately 4% of all ovarian neoplasms. While most ovarian fibromas are unilateral, approximately 10% can be bilateral, particularly in the context of Gorlin syndrome (1). In contrast, ovarian fibrosarcomas are almost exclusively unilateral (2). Although malignant transformation into fibrosarcoma is exceedingly rare, fibrosarcomas are characterized by elevated mitotic activity (typically ≥4/10 HPF), increased cellularity, diffuse moderate to severe nuclear atypia, often tumor necrosis/hemorrhage, and frequently display highly aggressive behavior with poor prognosis (3). Historically, distinguishing ovarian fibroma from fibrosarcoma has primarily depended on evaluating mitotic activity. It is noteworthy that certain cellular fibromas may show a high mitotic rate, but typically display only mild to moderate atypia, minimal or absent tumor necrosis, lack atypical mitoses, and manifest indolent clinical progression with favorable prognosis. Thus, they represent an intermediate pathological and biological state. The concept of “mitotically active cellular fibroma of the ovary” was introduced by Irving JA (4) and was later incorporated as a distinct entity in the WHO Classification of Female Genital Tumors (5). MACF is characterized by an elevated mitotic index (≥4/10 HPF) but only mild–moderate atypia and lacks necrosis, defining a rare ovarian tumor with low malignant potential, yet infrequent recurrences can arise. Accordingly, meticulous multidisciplinary pathological review is paramount for optimal management.

MACF has been reported across a broad age spectrum, from pediatric to postmenopausal patients, with a median age of 41 years (range: 14–93 years) (4, 6). Clinically, MACF most often presents as an asymptomatic adnexal mass discovered incidentally; a minority of patients may experience lower abdominal pain or symptoms secondary to mass effect, such as bladder compression. Imaging typically reveals a unilateral, well-demarcated solid ovarian mass (measuring 4.3-14.9cm), often exhibiting prominent vascularity. Tumor markers are generally nonspecific, although mild elevations in CA125 may occur; hormonal activity and disturbances of the menstrual cycle are typically absent (4, 6–8). Preoperative diagnosis remains challenging due to the lack of reliable serum tumor markers or characteristic imaging features. For solid ovarian masses, particularly those demonstrating significant vascularization, surgical exploration is recommended alongside evaluation systems such as Ovarian-Adnexal Reporting and Data System (O-RADS).

The differential diagnosis of a solid, vascular ovarian mass in a young woman is broad. Preoperatively, this includes benign entities such as fibroma/thecoma and pedunculated leiomyoma, as well as malignant tumors like granulosa cell tumors or sarcomas. On pathological examination, the primary differential for this lesion was between cellular fibroma, MACF, and fibrosarcoma. The high cellularity and mitotic rate excluded typical fibroma, while the lack of diffuse severe atypia and necrosis excluded fibrosarcoma.

Given the rarity of MACF, optimal management guidelines have not been clearly established. Nevertheless, most reported cases indicate excellent prognosis following conservative surgical management, with a low recurrence rate and prolonged survival (4, 6–9). Rare instances of local recurrence have been documented, especially in tumors with pronounced nuclear atypia, intraoperative rupture, or extensive adhesions (10). Due to the indolent nature of MACF, fertility-sparing surgery-typically unilateral salpingo-oophorectomy-is generally sufficient (4, 11). Preservation of the uterus may be considered on an individual basis. Conservative management is particularly suitable for younger women wishing to preserve fertility, given the tumor’s localized nature and intact ovarian surface. Laparoscopic excision utilizing containment bags to prevent intraperitoneal tumor dissemination is acceptable, and small series and case reports suggest laparoscopy is not associated with adverse oncologic outcomes (7, 8, 12); our case supports the feasibility of this approach and offers benefits of faster recovery. For larger tumors or when preoperative imaging suggests malignancy, open surgery is preferred to achieve complete resection and prevent tumor spillage. Regardless of the surgical approach, long-term follow-up is recommended for early detection of the rare possibility of recurrence.

A particularly challenging feature of this case was the markedly elevated Ki-67 index of 60%, which is higher than values typically reported for MACF and contributed significantly to the initial diagnostic uncertainty. The Ki-67 index is a powerful prognostic marker in epithelial ovarian cancer, where high values correlate strongly with aggressive behavior and poor survival (13). However, its prognostic value in ovarian sex cord-stromal tumors is not as well-established and can be discordant with morphology. While some studies suggest a correlation between a high Ki-67 index and recurrence in granulosa cell tumors (14), its significance in the fibroblastic series of tumors is poorly defined. This case highlights that in fibroblastic tumors, morphology—specifically the absence of severe atypia and necrosis—remains the cornerstone of diagnosis and should trump proliferative index alone in the differential between MACF and fibrosarcoma. This principle guided the intraoperative decision to proceed with conservative surgery, correctly prioritizing definitive histological features over an ambiguous proliferative marker.

## 4 Conclusion

MACF is a rare ovarian neoplasm where the primary clinical challenge is its pathological distinction from fibrosarcoma. This case powerfully demonstrates that a high mitotic rate and a markedly elevated Ki-67 index, while alarming, are not sufficient for a diagnosis of sarcoma in the absence of diffuse, severe nuclear atypia. Morphological criteria as defined by the WHO remain the gold standard. For young women, a fertility-sparing laparoscopic approach with meticulous surgical technique to ensure tumor containment is a safe and effective treatment.

## 5 Limitation

This report is limited by its single-case design and retrospective nature, which restrict generalizability. Whether larger tumors (>10cm) are suitable for laparoscopic surgery remains to be discussed. Furthermore, this case cannot be used to define a prognostic threshold for the Ki-67 index in MACF. It also highlights the known challenge that frozen section analysis for spindle cell neoplasms can be inconclusive. Although the 24-month course is favorable, longer-term follow-up and larger studies are necessary to definitively establish optimal management and recurrence risk for MACF.

## Data Availability

The original contributions presented in the study are included in the article/supplementary material. Further inquiries can be directed to the corresponding author.

## References

[1] Zhu M Li J Duan J Yang J Gu W Jiang W. Bilateral ovarian fibromas as the sole manifestation of Gorlin syndrome in a 22-year-old woman: a case report and literature review. Diagn Pathol. (2023) 18:118. doi: 10.1186/s13000-023-01406-9, PMID: 37907964 PMC10617060

[2] Sun TT Cheng NH Cao DY Peng P. Ovarian fibrosarcoma: A single-institution experience and a review of the literature. J Ovarian Res. (2020) 13:142. doi: 10.1186/s13048-020-00749-x, PMID: 33292402 PMC7724700

[3] Mraihi F Basly J Slama F Azouz E Ayari A Chelli D. Ovarian fibrosarcoma: Diagnostic challenges and treatment options, a case report. Int J Surg Case Rep. (2023) 112:108938. doi: 10.1016/j.ijscr.2023.108938, PMID: 37871372 PMC10667754

[4] Irving JA Alkushi A Young RH Clement PB. Cellular fibromas of the ovary: a study of 75 cases including 40 mitotically active tumors emphasizing their distinction from fibrosarcoma. Am J Surg Pathol. (2006) 30:929–38. doi: 10.1097/00000478-200608000-00001, PMID: 16861962

[5] Cree IA White VA Indave BI Lokuhetty D. Revising the WHO classification: female genital tract tumours. Histopathology. (2020) 76:151–6. doi: 10.1111/his.13977, PMID: 31846528

[6] Bi R Zhao Y Yang WT. Mitotically active cellular fibroma of ovary: a clinicopathologic analysis. Zhonghua Bing Li Xue Za Zhi. (2013) 42:660–4. doi: 10.3760/cma.j.issn.0529-5807.2013.10.004, PMID: 24433727

[7] Kim ET Song YJ Park KY Hwang CS Lee NK Roh HJ. Clinical, radiological, and pathological features of mitotically active cellular fibroma of ovary: A review of cases with literature review. Taiwan J Obstet Gynecol. (2024) 63:722–30. doi: 10.1016/j.tjog.2024.04.016, PMID: 39266154

[8] Kim JY Na K Kim HS. Clinicopathological characteristics of mitotically-active cellular fibroma of the ovary: a single-institutional experience. Anticancer Res. (2017) 37:2557–64. doi: 10.21873/anticanres.11599, PMID: 28476827

[9] Azari-Yam A Montazeri S Ashjaei B Sarmadi S. Ovarian mitotically active cellular fibroma of childhood: review of literature and report of a case. J Pediatr Hematol Oncol. (2025) 47:279–83. doi: 10.1097/MPH.0000000000003059, PMID: 40479607

[10] Olivadese R Ramponi A Boldorini R Dalla Dea G Palicelli A. Mitotically active cellular fibroma of the ovary recurring after the longest interval of time (16 yr): A challenging case with systematic literature review. Int J Gynecol Pathol. (2021) 40:441–7. doi: 10.1097/PGP.0000000000000731, PMID: 33252401

[11] Matsuda K Tateishi S Akazawa Y Kinoshita A Yoshida S Morisaki S. Rapid growth of mitotically active cellular fibroma of the ovary: a case report and review of the literature. Diagn Pathol. (2016) 11:101. doi: 10.1186/s13000-016-0554-7, PMID: 27770806 PMC5075201

[12] Yamada T Hattori K Satomi H Hirose Y Nakai G Daimon A. Mitotically active cellular fibroma of the ovary: a case report and literature review. J Ovarian Res. (2015) 8:65. doi: 10.1186/s13048-015-0191-x, PMID: 26437718 PMC4595272

[13] Qiu D Cai W Zhang Z Li H Zhou D. High Ki-67 expression is significantly associated with poor prognosis of ovarian cancer patients: evidence from a meta-analysis. Arch Gynecol Obstet. (2019) 299:1415–27. doi: 10.1007/s00404-019-05082-3, PMID: 30761416

[14] Bayram A Bagbudar S Yilmaz I Sozen H Minareci Y Altay AY. Predicting recurrence in adult granulosa cell tumors: the role of Ki67, p53, and TERT mutations. Arch Gynecol Obstet. (2025) 311:415–21. doi: 10.1007/s00404-024-07888-2, PMID: 39688684

